# Description of a new horned toad of *Megophrys* Kuhl & Van Hasselt, 1822 (Anura, Megophryidae) from southwest China

**DOI:** 10.3897/zookeys.974.56070

**Published:** 2020-10-07

**Authors:** Haijun Su, Shengchao Shi, Yanqing Wu, Guangrong Li, Xiaogang Yao, Bin Wang, Shize Li

**Affiliations:** 1 College of Forestry, Guizhou University, Guiyang 550002, China Guizhou University Guiyang China; 2 Chengdu Institute of Biology, Chinese Academy of Sciences, Chengdu 610041, China Chinese Academy of Sciences Chengdu China; 3 Nanjing Institute of Environmental Sciences, Ministry of Ecology and Environment of China, Nanjing 210042, China Nanjing Institute of Environmental Sciences Nanjing China; 4 Kuankuoshui National Nature Reserve Administration, Suiyang 563300, China Kuankuoshui National Nature Reserve Administration Suiyang China

**Keywords:** China, molecular phylogenetic analysis, morphology, new species, taxonomy

## Abstract

A new species of the genus *Megophrys* is described from Guizhou Province, China. Molecular phylogenetic analyses supported the new species as an independent clade nested into the *Megophrys*. The new species could be distinguished from its congeners by a combination of the following characters: body size moderate (SVL 49.3–58.2 mm in males); vomerine ridges present distinctly, vomerine teeth present; tongue feebly notched behind; tympanum distinctly visible, oval; two metacarpal tubercles in hand; toes with one-third webbing and wide lateral fringes; heels overlapped when thighs are positioned at right angles to the body; tibiotarsal articulation reaching the level between tympanum and eye when leg stretched forward; an internal single subgular vocal sac present in male; in breeding male, the nuptial pads with large and sparse black nuptial spines present on the dorsal bases of the first two fingers.

## Introduction

The toad genus *Megophrys* Kuhl & Van Hasselt, 1822 (Anura; Megophryidae) is widely distributed in eastern and central China, throughout southeastern Asia, and extending to the islands of the Sunda Shelf and the Philippines (Frost 2020). The taxonomic assignments of the taxa in this group have been debated for a long time (e.g., Tian and Hu 1983; Dubois 1987; Lathrop 1997; Rao and Yang 1997; Jiang et al. 2003; Delorme et al. 2006; Fei et al. 2009; Chen et al. 2016; Fei and Ye 2016; Deuti et al. 2017; Mahony et al. 2017; Frost 2020). Regardless, molecular phylogenetic studies indicate the group as a monophyletic group (Chen et al. 2017; Mahony et al. 2017; Li et al. 2018b; Liu et al. 2018, 2020; Wang et al. 2020), and thus some studies regarded it as a large genus, *Megophryssensu lato* (Mahony et al. 2017; Li et al. 2018b; Liu et al. 2018, 2020; Wang et al. 2020; Frost 2020) while other studies divided the taxon into different genera and subgenera (Chen et al. 2017; Fei and Ye 2016; Liu et al. 2018).

The genus *Megophrys* currently contains 106 species, of which 50 species have been described in the last decade (Frost 2020). Many cryptic species were indicated in the genus by molecular phylogenetic frameworks (Chen et al. 2017; Liu et al. 2018). In recent years, four species were described from Guizhou Province, China: *Megophrys liboensis* Zhang, Li, Xiao, Li ,Pan, Wang, Zhang & Zhou, 2017, *Megophrys leishanensis* Li, Xu, Liu, Jiang, Wei & Wang, 2018, *Megophrys jiangi* Liu, Li, Wei, Xu, Cheng, Wang & Wu, 2020, and *Megophrys chishuiensis* Xu, Li, Liu, Wei, & Wang, 2020. However, many areas have not been well investigated in this province, and it is expected that there are still cryptic species of the toads in the region.

During field surveys in the Huanglian Nature Reserve, Tongzi County, and Kuankuoshui National Nature Reserve, Suiyang County in Guizhou Province, China, we collected a number of *Megophrys* specimens. Molecular phylogenetic analyses, morphological comparisons, and bioacoustics data support it as an undescribed species.

## Materials and methods

### Sampling

**Figure 1.**
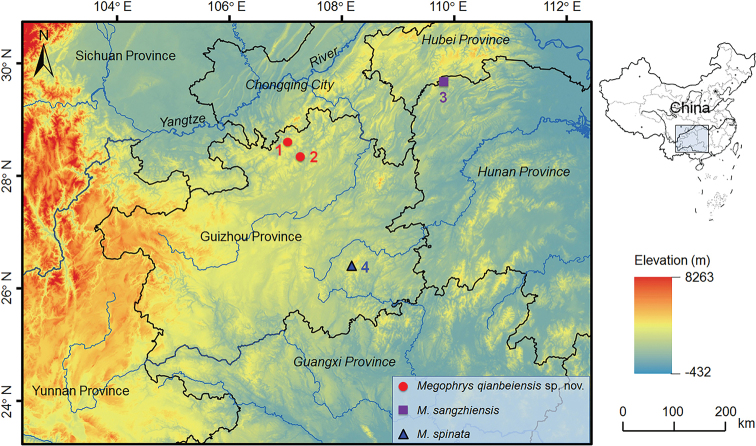
Sampling localities of *Megophrys qianbeiensis* sp. nov., *M. sangzhiensis* and *M. spinata* in China. 1. Huanglian Nature Reserve, Tongzi County, Guizhou Province; 2. Kuankuoshui National Nature Reserve, Suiyang County, Guizhou Province; 3. Badagong Mountain, Hunan Province, China; 4. Leigong Mountain, Guizhou Province.

A total of nine molecular samples were collected in this study: five adult males of the undescribed species from two localities of Guizhou Province, China, two *M. sangzhiensis* and two *M. spinata* (Table 1; Fig. 1). In the field, the toads were euthanized using isoflurane, and the specimens were fixed in 75 % ethanol. Tissue samples were taken and preserved separately in 99% ethanol prior to fixation. The specimens were deposited in Chengdu Institute of Biology, Chinese Academy of Sciences (**CIB**, **CAS**).

### Molecular data and phylogenetic analyses

Total DNA was extracted using a standard phenol-chloroform extraction protocol (Sambrook et al. 1989). Two fragments of the mitochondrial 16S rRNA (16S) and cytochromeoxidase subunit I (COI) genes were amplified. For 16S, the primers P7 (5’-CGCCTGTTTACCAAAAACAT-3’) and P8 (5’-CCGGTCTGAACTCAGATCACGT-3’) were used following Simon et al. (1994), and for COI, Chmf4 (5’-TYTCWACWAAYCAYAAAGAYATCGG-3’) and Chmr4 (5’-ACYTCRGGRTGRCCRAARAATCA-3’) were used following Che et al. (2012). Gene fragments were amplified under the following conditions: an initial denaturing step at 95 °C for 4 min; 36 cycles of denaturing at 95 °C for 30 s, annealing at 52 °C (for 16S)/47 °C (for COI) for 40 s and extending at 72 °C for 70 s. Sequencing was conducted using an ABI3730 automated DNA sequencer in Shanghai DNA BioTechnologies Co., Ltd. (Shanghai, China). New sequences were deposited in GenBank (for accession numbers see Table 1).

**Table 1.** Information for samples used in molecular phylogenetic analyses in this study.

ID	Species	Voucher number	Locality	GenBank accession number	
				16S	COI
1	*Megophrys qianbeiensis* sp. nov.	CIBTZ20190608015	Huanglian Nature Reserve, Guizhou, China	MT651553	MT654520
2	*Megophrys qianbeiensis* sp. nov.	CIBTZ20190608017	Huanglian Nature Reserve, Guizhou, China	MT651554	MT654521
3	*Megophrys qianbeiensis* sp. nov.	CIBTZ20160715003	Huanglian Nature Reserve, Guizhou, China	MT651555	MT654522
4	*Megophrys qianbeiensis* sp. nov.	CIBKKS20180722002	Huanglian Nature Reserve, Guizhou, China	MT651556	MT654523
5	*Megophrys qianbeiensis* sp. nov.	CIBKKS20180722001	Kuankuoshui Nature Reserve, Guizhou, China	MT651557	MT654524
6	*Megophrys sangzhiensis*	CIBSZ2012062005	Badagongshan Nature Reserve, Hunan, China	MT651558	MT654525
7	*Megophrys sangzhiensis*	CIBSZ2012062008	Badagongshan Nature Reserve, Hunan, China	MT651559	MT654526
8	*Megophrys sangzhiensis*	SYSa004307	Zhangjiajie, Hunan, China	MH406798	MH406260
9	*Megophrys spinata*	CIBLS20190801001	Leigong Shan, Guizhou, China	MT651551	MT654518
10	*Megophrys spinata*	CIBLS20190801002	Leigong Shan, Guizhou, China	MT651552	MT654519
11	*Megophrys spinata*	SYSa002227	Leigong Shan, Guizhou, China	MH406676	MH406116
12	*Megophrys binlingensis*	KIZ025807	Wawu Shan, Sichuan, China	KX811852	KX812115
13	*Megophrys binlingensis*	SYSa005313	Wawu Shan, Sichuan, China	MH406892	MH406354
14	*Megophrys binlingensis*	SYSa005314	Wawu Shan, Sichuan, China	MH406893	MH406355
15	*Megophrys binchuanensis*	KIZ019441	Jizu Shan, Yunnan, China	KX811849	KX812112
16	*Megophrys palpebralespinosa*	KIZ011603	Pu Hu Nature Reserve, Thanh Hoa, Vietnam	KX811888	KX812137
17	*Megophrys omeimontis*	KIZ025765	Emei Shan, Sichuan, China	KX811884	KX812136
18	*Megophrys angka*	KIZ040591	Kiew Mae Pan nature trail, Chiang Mai, Thailand	MN508052	–
19	*Megophrys wuliangshanensis*	KIZ046812	Huangcaoling, Yunnan, China	KX811881	KX812129
20	*Megophrys daweimontis*	KIZ048997	Dawei Shan, Yunnan, China	KX811867	KX812125
21	*Megophrys jingdongensis*	KIZ-LC0805067	Huanglianshan National Nature Reserve, Yunnan, China	KX811872	KX812131
22	*Megophrys fansipanensis*	VNMN 2018.01	Lao Cai, Sa Pa, Vietnam	MH514886	–
23	*Megophrys hoanglienensis*	VNMN 2018.02	Lao Cai, Sa Pa, Vietnam	MH514889	–
24	*Megophrys minor*	KIZ01939	Qingcheng Shan, Sichuan, China	KX811896	KX812145
25	*Megophrys jiangi*	CIBKKS20180722006	Kuankuosui Nature Reserve, Guizhou, China	MN107743	MN107748
26	*Megophrys chishuiensis*	CIBCS20190518031	Chishui Nature Reserve, Guizhou, China	MN954707	MN928958
27	*Megophrys dongguanensis*	SYS a001972	Yinping Shan, Guangdong, China	MK524098	MK524129
28	*Megophrys nankunensis*	SYS a004498	Nankun Shan, Guangdong, China	MK524108	MK524139
29	*Megophrys cheni*	SYS a001427	Jinggang Shan, Jiangxi, China	KJ560391	–
30	*Megophrys obesa*	SYS a002272	Heishiding Nature Reserve, Guangdong, China	KJ579122	–
31	*Megophrys ombrophila*	KRM18	Wuyishan, Fujian, China	KX856404	–
32	*Megophrys wugongensis*	SYS a002610	Wugongshan Scenic Area, Jiangxi, China	MK524114	MK524145
33	*Megophrys lini*	SYS a002370	Suichuan, Jiangxi, China	KJ560412	–
34	*Megophrys xiangnanensis*	SYS a002874	Yangming Shan, Hunan, China	MH406713	MH406165
35	*Megophrys nanlingensis*	SYS a001959	Nanling Nature Reserve, Guangdong, China	MK524111	MK524142
36	*Megophrys kuatunensis*	SYS a001579	Wuyi Shan, Fujian, China	KJ560376	–
37	*Megophrys jinggangensis*	KIZ07132	Chashan Forest Farm, Jiangxi, China	KX811840	KX812108
38	*Megophrys lishuiensis*	WYF00169	Lishui, Zhejiang, China	KY021418	–
39	*Megophrys xianjuensis*	CIBXJ190505	Xianju, Zhejiang, China	MN563753	MN563769
40	*Megophrys wushanensis*	KIZ045469	Guangwu Shan, Sichuan, China	KX811838	KX812094
41	*Megophrys baolongensis*	KIZ019216	Baolong, Chongqing, China	KX811813	KX812093
42	*Megophrys leishanensis*	CIBLS20171101001	Leigong Shan, Guizhou, China	MK005310	MK005306
43	*Megophrys yangmingensis*	SYS a002877	Yangming Shan, Hunan, China	MH406716	MH406168
44	*Megophrys shimentaina*	SYS a002077	Shimentai Nature Reserve, Guangdong, China	MH406655	MH406092
45	*Megophrys jiulianensis*	SYS a002107	Jiulian Shan, Jiangxi, China	MK524099	MK524130
46	*Megophrys shunhuangensis*	HNNU16SH02	Shunhuang Mountains, Hunan, China	MK836037	–
47	*Megophrys mirabilis*	SYS a002192	Huaping Nature Reserve, Guangxi, China	MH406669	MH406109
48	*Megophrys tuberogranulata*	Tissue ID: YPX10987	Badagongshan Nature Reserve, Hunan, China	KX811823	KX812095
49	*Megophrys huangshanensis*	KIZ022004	Huang Shan, Anhui, China	KX811821	KX812107
50	*Megophrys boettgeri*	Tissue ID: YPXJK033	Wuyi Shan, Fujian, China	KX811814	KX812104
51	*Megophrys liboensis*	GNUG:20160408003	Libo, Guizhou, China	MF285262	–
52	*Megophrys mufumontana*	SYS a006391	Mufu Shan, Hunan, China	MK524105	MK524136
53	*Megophrys brachykolos*	ROM 16634	Hong Kong, China	KX811897	KX812150
54	*Megophrys acuta*	SYS a001957	Heishiding Nature Reserve, Guangdong, China	KJ579118	–
55	*Megophrys gerti*	ITBCZ 1108	Nui Chua National Park, Ninh Thuan, Vietnam	KX811917	KX812161
56	*Megophrys elfina*	ZMMU ABV-00454	Bidoup Mountain, Lam Dong, Vietnam	KY425379	–
57	*Megophrys synoria*	FMNH 262778	O’Reang, Mondolkiri, Cambodia	KY022198	–
58	*Megophrys hansi*	KIZ010360	Phong Dien Nature Reserve, Thua Thien Hue, Vietnam	KX811913	KX812155
59	*Megophrys microstoma*	KIZ048799	Xiaoqiaogou Nature Reserve, Yunnan, China	KX811914	KX812156
60	*Megophrys pachyproctus*	KIZ010978	Beibeng, Xizang, China	KX811908	KX812153
61	*Megophrys baluensis*	ZMH A13125	Gunung Kinabalu National Park, Kogopan Trail, Malaysia	KJ831310	–
62	*Megophrys stejnegeri*	KU 314303	Pasonanca Natural Park, Zamboanga, Philippines	KX811922	KX812052
63	*Megophrys ligayae*	ZMMU NAP-05015	Palawan, Philippines	KX811919	KX812051
64	*Megophrys kobayashii*	UNIMAS 8148	Gunung Kinabalu National Park, Sabah, Malaysia	KJ831313	–
65	*Megophrys nasuta*	KIZ019419	Malaysia	KX811921	KX812054
66	*Megophrys edwardinae*	FMNH 273694	Bintulu, Sarawak, Malaysia	KX811918	KX812050
67	*Megophrys aceras*	KIZ025467	Khao Nan National Park, Nakhon Si Thammarat, Thailand	KX811925	KX812159
68	*Megophrys dringi*	UNIMAS 8943	Gunung Mulu National Park, Sarawak, Malaysia	KJ831317	–
69	*Megophrys maosonensis*	KIZ016045	Xiaoqiaogou Nature Reserve, Yunnan, China	KX811780	KX812080
70	*Megophrys mangshanensis*	KIZ021786	Nanling National Forest Park, Guangdong, China	KX811790	KX812079
71	*Megophrys flavipunctata*	SDBDU2009.297	East Khasi Hills dist., Meghalaya	KY022307	MH647536
72	*Megophrys glandulosa*	KIZ048439	Husa, Yunnan, China	KX811762	KX812075
73	*Megophrys medogensis*	KIZ06621	Beibeng, Xizang, China	KX811767	KX812082
74	*Megophrys periosa*	BNHS 6061	West Kameng dist., Arunachal Pradesh, IN	KY022309	MH647528
75	*Megophrys himalayana*	SDBDU2009.75	East Siang dist., Arunachal Pradesh, IN	KY022311	–
76	*Megophrys sanu*	K5198/ZSI11393	–	KX894679	–
77	*Megophrys zhangi*	KIZ014278	Zhangmu, Xizang, China	KX811765	KX812084
78	*Megophrys katabhako*	ZSIA11799	–	KX894669	–
79	*Megophrys major*	SYSa002961	Zhushihe, Yunnan, China	MH406728	MH406180
80	*Megophrys oreocrypta*	BNHS 6046	West Garo Hills dist., Meghalaya	KY022306	–
81	*Megophrys auralensis*	NCSM 79599	Aural, Kampong Speu, Cambodia	KX811807	–
82	*Megophrys parva*	SYSa003042	Zhushihe, Yunnan, China	MH406737	MH406189
83	*Megophrys nankiangensis*	CIB ZYC517	Nanjiang, Sichuan, China	KX811900	–
84	*Megophrys wawuensis*	KIZ025799	Wawu Shan, Sichuan, China	KX811902	KX812062
85	*Megophrys gigantica*	SYSa003933	Wuliang shan, Yunnan, China	MH406775	MH406235
86	*Megophrys shapingensis*	KIZ014512	Liziping Nature Reserve, Sichuan, China	KX811904	KX812060
87	*Megophrys montana*	LSUMZ 81916	Sukabumi, Java, Indonesia	KX811927	KX812163
88	*Megophrys lancip*	MZB:Amp:22233	–	KY679891	–
89	*Megophrys feae*	KIZ046706	Huangcaoling, Yunnan, China	KX811810	KX812056
90	*Megophrys chuannanensis*	CIB20050081	Hejiang, Sichuan, China	KM504261	–
91	*Megophrys carinense*	Tissue ID: YPX20455	Dayao Shan, Guangxi, China	KX811811	KX812057
92	*Megophrys popei*	SYS a000589	Naling Nature Reserve, Guangdong, China	KM504251	–
93	*Megophrys intermedia*	ZFMK 87596	U Bo, Phong Nha-Ke Bang NP, Vietnam	HQ588950	–
94	*Leptobrachium boringii*	Tissue ID: YPX37539	Emei Shan, Sichuan, China	KX811930	KX812164
95	*Leptobrachella oshanensis*	KIZ025778	Emei Shan, Sichuan, China	KX811928	KX812166

For molecular analyses, the available sequence data for congeners of *Megophrys* were downloaded from GenBank (Table 1), primarily from previous studies (Chen et al. 2017; Liu et al. 2018). For phylogenetic analyses, corresponding sequences of one *Leptobrachella oshanensis* (Liu, 1950) and one *Leptobrachium boringii* (Liu, 1945) were also downloaded (Table 1), and used as outgroups according to Mahony et al. (2017). Sequences were assembled and aligned using the Clustalw module in BioEdit v.7.0.9.0 (Hall 1999) with default settings. Alignments were checked by eye and revised manually if necessary. For phylogenetic analyses of mitochondrial DNA, the dataset concatenated with 16S and COI gene sequences. To avoid under- or over-parameterization (Lemmon and Moriarty 2004; McGuire et al. 2007), the best partition scheme and the best evolutionary model for each partition were chosen for the phylogenetic analyses using PARTITIONFINDER v. 1.1.1 (Robert et al. 2012). In this analysis, 16S gene and each codon position of COI gene were defined, and Bayesian Inference Criteria was used. As a result, the analysis suggested that the best partition scheme is16S gene/each codon position of COI gene, and selected GTR + G + I model as the best model for each partition. Phylogenetic analyses were conducted using maximum likelihood (ML) and Bayesian Inference (BI) methods, implemented in PhyML v. 3.0 (Guindon et al. 2010) and MrBayes v. 3.12 (Ronquist and Huelsenbeck 2003), respectively. For the ML tree, branch supports were drawn from 10,000 nonparametric bootstrap replicates. In BI, two runs each with four Markov chains were simultaneously run for 50 million generations with sampling every 1,000 generations. The first 25 % trees were removed as the “burn-in” stage followed by calculations of Bayesian posterior probabilities and the 50% majority-rule consensus of the post burn-in trees sampled at stationarity.

### Morphological comparisons

In total, 16 specimens including six males of the undescribed species, five males of *M. sangzhiensis*, and five males of *M. spinata* were measured (for voucher information see Table 2). The terminology and methods followed Fei et al. (2009). Measurements were taken with a dial caliper to 0.1 mm. Twenty-one morphometric characters of adult specimens were measured:

**ED** eye diameter (distance from the anterior corner to the posterior corner of the eye);

**FL** foot length (distance from tarsus to the tip of fourth toe);

**HDL** head length (distance from the tip of the snout to the articulation of jaw);

**HDW** maximum head width (greatest width between the left and right articulations of jaw);

**HLL** hindlimb length (maximum length from the vent to the distal tip of the Toe IV);

**IAE** distance between posterior corner of eyes;

**IFE** distance between anterior corner of eyes;

**IND** internasal distance (minimum distance between the inner margins of the external nares);

**IOD** interorbital distance (minimum distance between the inner edges of the upper eyelids);

**LAL** length of lower arm and hand (distance from the elbow to the distal end of the Finger IV);

**LW** lower arm width (maximum width of the lower arm);

**NED** nasal to eye distance (distance between the nasal and the anterior corner of the eye);

**NSD** nasal to snout distance (distance between the nasal the posterior edge of the vent);

**SVL** snout-vent length (distance from the tip of the snout to the posterior edge of the vent);

**SL** snout length (distance from the tip of the snout to the anterior corner of the eye);

**TFL** length of foot and tarsus (distance from the tibiotarsal articulation to the distal end of the Toe IV);

**THL** thigh length (distance from vent to knee);

**TL** tibia length (distance from knee to tarsus);

**TW** maximal tibia width;

**TYD** maximal tympanum diameter;

**UEW** upper eyelid width (greatest width of the upper eyelid margins measured perpendicular to the anterior-posterior axis).

**Table 2.** Measurements of the adult specimens of *Megophrys qianbeiensis* sp. nov., *M. spinata*, and *M. sangzhiensis*. Units are given in mm. See abbreviations for the morphological characters in Materials and methods section.

Species	Voucher number	Sex	SVL	HDL	HDW	SL	NED	NSD	IND	IOD	ED	UEW	LAL	LW	HLL	THL	TL	TW	TFL	FL	TYD	IFE	IAE
*Megophrys qianbeiensis* sp. nov.	CIBTZ20190608016	male	58.2	16.5	21.0	6.7	2.7	3.9	7.5	5.1	6.9	6.2	25.0	6.7	89.3	27.9	32.4	8.2	40.7	28.2	4.3	10.1	16.4
*Megophrys qianbeiensis* sp. nov.	CIBTZ20190608018	male	55.1	14.9	20.6	6.9	3.0	4.0	7.0	4.2	6.5	5.1	25.0	6.5	93.0	28.4	30.6	8.7	44.0	29.1	3.5	9.0	16.0
*Megophrys qianbeiensis* sp. nov.	CIBTZ20190608017	male	56.3	14.6	19.2	7.2	3.2	3.6	6.7	4.3	6.5	5.3	24.0	6.9	87.0	27.6	28.0	7.8	38.3	25.7	3.2	10.9	15.0
*Megophrys qianbeiensis* sp. nov.	CIBTZ20160715003	male	54.1	17.0	20.8	6.9	3.4	3.7	6.3	4.5	6.9	6.0	25.4	5.7	93.7	27.0	30.8	7.8	43.7	29.4	3.5	10.4	15.8
*Megophrys qianbeiensis* sp. nov.	CIBTZ20190608015	male	52.6	15.3	19.4	6.8	2.4	4.3	6.9	3.7	5.9	5.5	24.1	7.4	86.9	24.1	28.3	8.3	41.4	28.0	3.3	10.0	15.7
*Megophrys qianbeiensis* sp. nov.	CIBKKS20180722001	male	49.3	15.5	18.3	6.8	3.0	3.5	5.7	5.3	5.4	5.3	20.4	6.6	76.9	24.5	25.0	7.0	34.5	24.5	3.4	7.8	14.0
*M. spinata*	CIBLS20190801002	male	56.2	14.9	18.4	6.0	3.0	3.4	5.8	4.2	5.1	6.1	24.2	5.5	93.7	27.4	29.9	6.1	40.8	28.6	2.7	9.1	14.0
*M. spinata*	CIBLS20190801004	male	53.5	14.5	19.1	7.1	2.8	4.1	6.0	5.0	5.7	5.0	24.1	5.9	99.0	29.8	30.4	8.0	43.1	28.1	2.8	9.6	14.4
*M. spinata*	CIBLS20190801001	male	54.8	14.6	18.6	6.7	2.8	3.9	6.0	4.8	5.8	4.5	24.3	6.1	87.7	27.6	28.9	7.0	39.7	26.4	2.8	9.2	14.2
*M. spinata*	CIBLS20190801003	male	51.2	14.3	18.8	6.6	2.9	3.6	6.1	5.2	6.0	5.1	25.1	6.5	93.0	26.1	29.6	7.7	41.6	29.6	2.5	9.1	14.0
*M. spinata*	CIBLS20160610008	male	53.8	15.8	18.4	5.7	2.7	3.8	6.3	5.3	5.5	4.5	24.0	7.4	85.7	26.9	29.0	6.0	39.2	27.9	2.9	8.5	14.3
*M. sangzhiensis*	CIBSZ2012062005	male	59.8	17.8	20.6	7.2	3.1	4.1	7.3	4.8	7.1	5.7	26.6	6.6	105.0	31.6	32.6	7.8	46.1	29.4	3.1	10.4	16.1
*M. sangzhiensis*	CIBSZ2012062008	male	58.8	17.8	21.5	7.6	3.0	4.4	7.3	4.7	7.4	6.1	26.8	6.2	97.3	30.3	31.6	7.8	42.9	26.7	3.4	10.9	17.3
*M. sangzhiensis*	CIBSZ2012062006	male	59.5	16.1	21.0	8.2	3.5	4.6	7.7	5.0	6.7	6.1	26.6	6.3	99.8	27.9	32.3	7.3	43.8	29.2	3.4	10.1	17.3
*M. sangzhiensis*	CIBSZ2012062019	male	57.4	18.0	20.9	7.3	3.5	3.7	6.8	5.0	6.4	5.1	26.2	6.1	99.5	30.2	32.6	8.1	43.2	29.9	3.7	10.4	17.3
*M. sangzhiensis*	CIBSZ2012062007	male	56.1	16.1	20.0	6.6	3.9	4.1	6.9	5.8	6.1	5.7	28.2	6.7	100.0	28.0	32.0	7.5	44.9	29.4	3.4	9.4	16.2

In order to reduce the impact of allometry, the correct value from the ratio of each character to SVL was calculated, and then was log-transformed for subsequent morphometric analyses. One-way analysis of variance (ANOVA) was used to test the significance of differences on morphometric characters between different species. The significance level was set at 0.05. To show the spatial distribution of different species on the morphometric characters, principal component analyses (PCA) were performed. These analyses were carried out in the R (R Development Core Team 2008). The new species was also compared with all other *Megophrys* species on morphology. Comparative data were obtained from related species as described in literature (Table 3).

**Table 3.** References for morphological characters for congeners of the genus *Megophrys*.

Species	Literature
*M. aceras* Boulenger, 1903	Boulenger 1903
*M. acuta* Wang, Li & Jin, 2014	Li et al. 2014
*M. ancrae* Mahony, Teeling & Biju, 2013	Mahony et al. 2013
*M. angka* Wu, Suwannapoom, Poyarkov, Chen, Pawangkhanant, Xu, Jin, Murphy & Che, 2019	Wu et al. 2019
*M. auralensis* Ohler, Swan & Daltry, 2002	Ohler et al. 2002
*M. baluensis* (Boulenger, 1899)	Boulenger 1899a
*M. baolongensis* Ye, Fei & Xie, 2007	Ye et al. 2007
*M. binchuanensis* Ye & Fei, 1995	Ye and Fei 1995
*M. binlingensis* Jiang, Fei & Ye, 2009	Fei et al. 2009
*M. boettgeri* (Boulenger, 1899)	Boulenger 1899b
*M. brachykolos* Inger & Romer, 1961	Inger and Romer 1961
*M. carinense* (Boulenger, 1889)	Boulenger 1889
*M. caobangensis* Nguyen, Pham, Nguyen, Luong, & Ziegler, 2020	Nguyen et al. 2020
*M. caudoprocta* Shen, 1994	Shen. 1994
*M. cheni* (Wang & Liu, 2014)	Wang et al. 2014
*M. chishuiensis* Xu, Li, Liu, Wei & Wang, 2020	Xu et al. 2020
*M. chuannanensis* (Fei, Ye & Huang, 2001)	Fei et al. 2001
*M. damrei* Mahony, 2011	Mahony 2011
*M. daweimontis* Rao & Yang, 1997	Rao and Yang 1997
*M. dongguanensis* Wang & Wang, 2019	Wang et al. 2019b
*M. dringi* Inger, Stuebing & Tan, 1995	Inger et al. 1995
*M. edwardinae* Inger, 1989	Inger 1989
*M. elfina* Poyarkov, Duong, Orlov, Gogoleva, Vassilieva, Nguyen, Nguyen, Nguyen, Che & Mahony, 2017	Poyarkov et al. 2017
*M. fansipanensis* Tapley, Cutajar, Mahony, Nguyen, Dau, Luong, Le, Nguyen, Nguyen, Portway, Luong & Rowley, 2018	Tapley et al. 2018
*M. feae* Boulenger, 1887	Boulenger 1887
*M. feii* Yang, Wang & Wang, 2018	Yang et al. 2018
*M. flavipunctata* Mahony, Kamei, Teeling & Biju, 2018	Mahony et al. 2018
*M. gerti* (Ohler, 2003)	Ohler 2003
*M. gigantica* Liu, Hu & Yang, 1960	Liu et al. 1960
*M. glandulosa* Fei, Ye & Huang, 1990	Fei et al. 1990
*M. hansi* (Ohler, 2003)	Ohler 2003
*M. himalayana* Mahony, Kamei, Teeling & Biju, 2018	Mahony et al. 2018
*M. hoanglienensis* Tapley, Cutajar, Mahony, Nguyen, Dau, Luong, Le, Nguyen, Nguyen, Portway, Luong & Rowley, 2018	Tapley et al. 2018
*M. huangshanensis* Fei & Ye, 2005	Fei and Ye 2005
*M. insularis* (Wang, Liu, Lyu, Zeng & Wang, 2017)	Wang et al. 2017a
*M. intermedia* Smith, 1921	Smith 1921
*M. jiangi* Liu, Li, Wei, Xu, Cheng, Wang & Wu, 2020	Liu et al. 2020
*M. jingdongensis* Fei & Ye, 1983	Fei et al. 1983
*M. jinggangensis* (Wang, 2012)	Wang et al. 2012
*M. jiulianensis* Wang, Zeng, Lyu & Wang, 2019	Wang et al. 2019b
*M. kalimantanensis* Munir, Hamidy, Matsui, Iskandar, Sidik & Shimada, 2019	Munir et al. 2019
*M. kobayashii* Malkmus & Matsui, 1997	Malkmus and Matsui 1997
*M. koui* Mahony, Foley, Biju & Teeling, 2017	Mahony et al. 2017
*M. kuatunensis* Pope, 1929	Pope 1929
*M. lancip* Munir, Hamidy, Farajallah & Smith, 2018	Munir et al. 2018
*M. leishanensis* Li, Xu, Liu, Jiang, Wei & Wang, 2018	Li et al. 2018b
*M. lekaguli* Stuart, Chuaynkern, Chan-ard & Inger, 2006	Stuart et al. 2006
*M. liboensis* (Zhang, Li, Xiao, Li, Pan, Wang, Zhang & Zhou, 2017)	Zhang et al. 2017
*M. ligayae* Taylor, 1920	Taylor 1920
*M. lini* (Wang & Yang, 2014)	Wang et al. 2014
*M. lishuiensis* (Wang, Liu & Jiang, 2017)	Wang et al. 2017b
*M. longipes* Boulenger, 1886	Boulenger 1886
*M. major* Boulenger, 1908	Boulenger 1908
*M. mangshanensis* Fei & Ye, 1990	Fei et al. 2012
*M. maosonensis* Bourret, 1937	Bourret 1937
*M. medogensis* Fei, Ye & Huang, 1983	Fei et al. 1983
*M. megacephala* Mahony, Sengupta, Kamei & Biju, 2011	Mahony et al. 2011
*M. microstoma* (Boulenger, 1903)	Boulenger 1903
*M. minor* Stejneger, 1926	Stejneger 1926
*M. mirabilis* Lyu, Wang & Zhao, 2020	Lyu et al. 2020
*M. montana* Kuhl & Van Hasselt, 1822	Kuhl and Van Hasselt 1822
*M. monticola* (Günther, 1864)	Günther 1864
*M. mufumontana* Wang, Lyu & Wang, 2019	Wang et al. 2019b
*M. nankiangensis* Liu & Hu, 1966	Hu and Liu 1966
*M. nankunensis* Wang, Zeng & Wang, 2019	Wang et al. 2019b
*M. nanlingensis* Lyu, Wang, Liu & Wang, 2019	Wang et al. 2019b
*M. nasuta* (Schlegel, 1858)	Schlegel 1858
*M. obesa* Wang, Li & Zhao, 2014	Wang et al. 2014
*M. ombrophila* Messenger & Dahn, 2019	Messenger et al. 2019
*M. omeimontis* Liu, 1950	Liu 1950
*M. oreocrypta* Mahony, Kamei, Teeling & Biju, 2018	Mahony et al. 2018
*M. oropedion* Mahony, Teeling & Biju, 2013	Mahony et al. 2013
*M. orientalis* Li, Lyu, Wang & Wang, 2020	Li et al. 2020
*M. pachyproctus* Huang, 1981	Huang and Fei 1981
*M. palpebralespinosa* Bourret, 1937	Bourret 1937
*M. parallela* Inger & Iskandar, 2005	Inger and Iskandar 2005
*M. parva* (Boulenger, 1893)	Boulenger 1893
*M. periosa* Mahony, Kamei, Teeling & Biju, 2018	Mahony et al. 2018
*M. popei* (Zhao, Yang, Chen, Chen & Wang, 2014)	Zhao et al. 2014
*M. robusta* Boulenger, 1908	Boulenger 1908
*M. rubrimera* Tapley, Cutajar, Mahony, Chung, Dau, Nguyen, Luong & Rowley, 2017	Tapley et al. 2017
*M. sangzhiensis* Jiang, Ye & Fei, 2008	Jiang et al. 2008
*M. serchhipii* (Mathew & Sen, 2007)	Mathew and Sen 2007
*M. shapingensis* Liu, 1950	Liu 1950
*M. shimentaina* Lyu, Liu & Wang, 2020	Lyu et al. 2020
*M. shuichengensis* Tian & Sun, 1995	Tian and Sun 1995
*M. shunhuangensis* Wang, Deng, Liu, Wu & Liu, 2019	Wang et al. 2019a
*M. spinata* Liu & Hu, 1973	Hu et al. 1973
*M. stejnegeri* Taylor, 1920	Taylor 1920
*M. synoria* (Stuart, Sok & Neang, 2006)	Stuart et al. 2006
*M. takensis* Mahony, 2011	Mahony 2011
*M. tuberogranulata* Shen, Mo & Li, 2010	Mo et al. 2010
*M. vegrandis* Mahony, Teeling, Biju, 2013	Mahony et al. 2013
*M. wawuensis* Fei, Jiang & Zheng, 2001	Fei et al. 2012
*M. wugongensis* Wang, Lyu & Wang, 2019	Wang et al. 2019b
*M. wuliangshanensis* Ye & Fei, 1995	Ye and Fei 1995
*M. wushanensis* Ye & Fei, 1995	Ye and Fei 1995
*M. xianjuensis* Wang, Wu, Peng, Shi, Lu & Wu, 2020	Wang et al. 2020
*M. xiangnanensis* Lyu, Zeng & Wang, 2020	Lyu et al. 2020
*M. yangmingensis* Lyu, Zeng & Wang, 2020	Lyu et al. 2020
*M. zhangi* Ye & Fei, 1992	Ye and Fei 2012
*M. zunhebotoensis* (Mathew & Sen, 2007)	Mathew and Sen 2007

### Bioacoustics analyses

The advertisement calls of the undescribed species were recorded from the holotype specimen CIBTZ20190608017 in a stream at ambient air temperature of 20.5 °C and air humidity of 87 % in the field on 8 June 2019 in Huanglian Nature Reserve, Tongzi County, Guizhou Province, China. The advertisement calls of *M. sangzhiensis* were recorded from the specimen CIBSZ2012062005 in a stream at ambient air temperature of 18.5 °C and air humidity of 85 % in the field on 20 June 2012 in Sangzhi County, Hunan Province, China. The advertisement calls of *M. spinata* were recorded from the specimen CIBLS20190801001 in a stream at ambient air temperature of 19.0 °C and air humidity of 85 % in the field on 1 August 2019 in Leishan County, Guizhou Province, China. SONY PCM-D50 digital sound recorder was used to record within 20 cm of the calling individual. The sound files in wave format were resampled at 48 kHz with sampling depth 24 bits. The sonograms and waveforms were generated by WaveSurfer software (Sjöander and Beskow 2000) from which all parameters and characters were measured. Ambient temperature was taken by a digital hygrothermograph.

## Results

### Phylogenetic analyses

**Figure 2.**
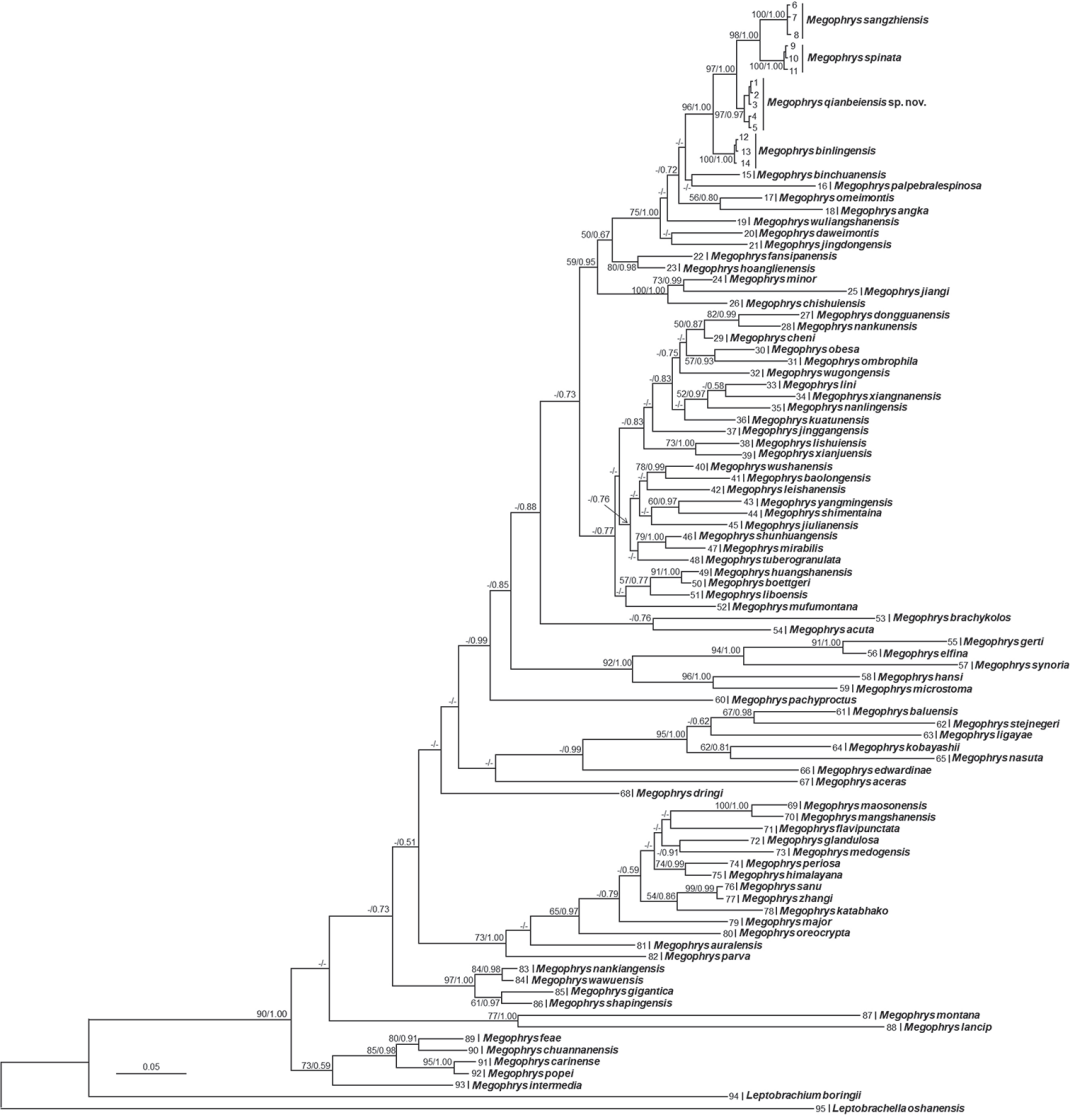
Bayesian Inference (BI) tree of the genus *Megophrys* reconstructed based on the 16S rRNA and COI gene sequences. Bayesian posterior probability resulted from BI analyses/ML bootstrap supports from Maximum Likelihood analyses were denoted beside each node. Samples 1–90 refer to Table 1.

Aligned sequence matrix of 16S+COI contains 1104 bp. ML and BI trees had almost consistent topology though relationships of some lineages were unresolved (Fig. 2). In trees, the undescribed species was clustered as an independent clade and sister to a clade in comprising of *M. sangzhiensis* and *M. spinata* (Fig. 2).

Genetic distances on COI gene with uncorrected *p*-distance model between all samples of the undescribed species were below 0.2%. The genetic distance between the undescribed species and its closest related species *M. sangzhiensis* was 4.3 % on COI gene, which was higher or at the same level with those among many pairs of congeners, for example, 3.6 % between *M. spinata* and *M. sangzhiensis*, 1.8% between *M. huangshanensis* and *M. boettgeri*, and 4.3 % between *M. maosonensis* and *M. mangshanensis* (Suppl. material 1: Table S1).

### Morphological comparisons

**Figure 3.**
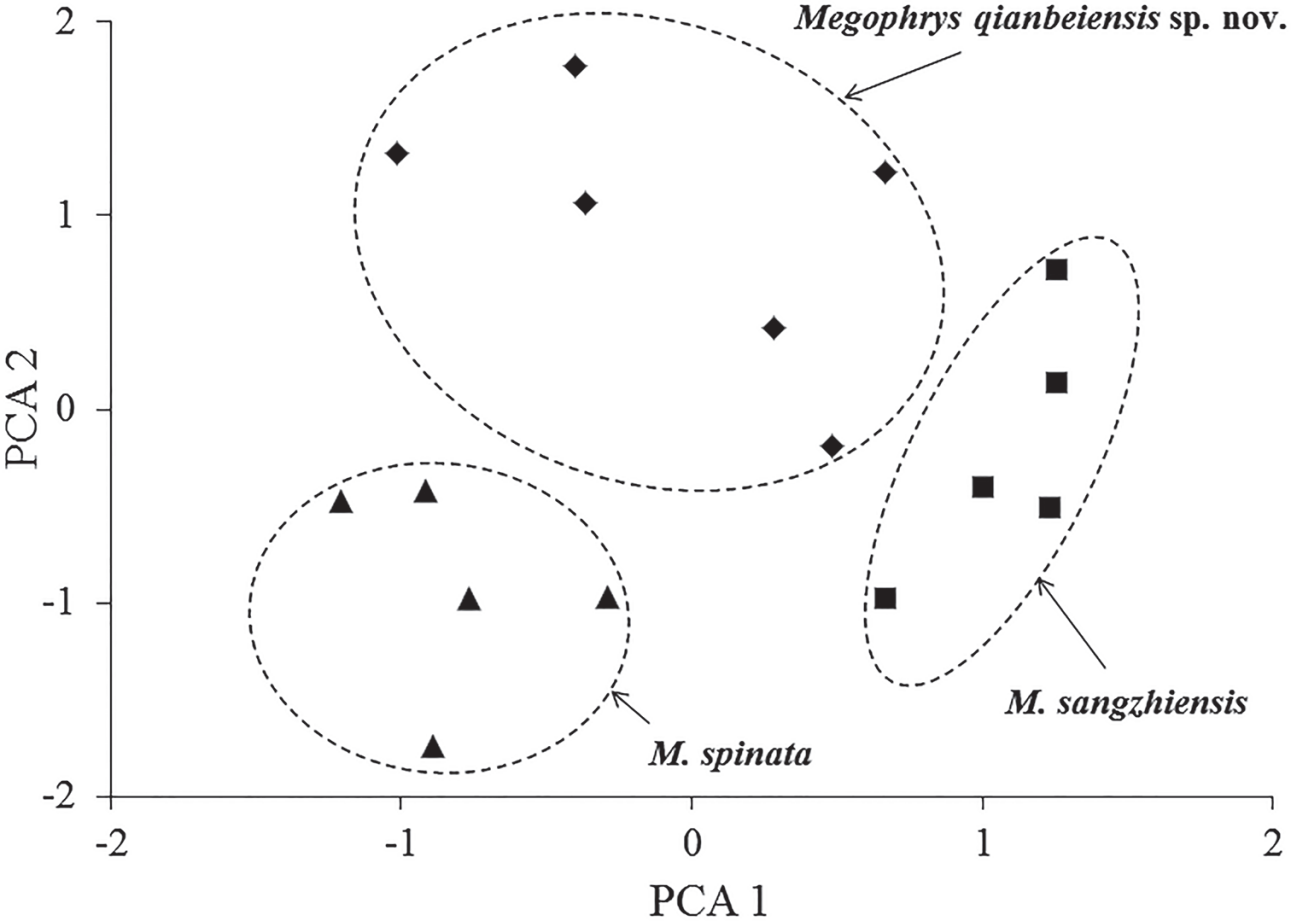
Plots of the first principal component (PCA1) versus the second (PCA2) for *Megophrys qianbeiensis* sp. nov., *M. sangzhiensis*, and *M. spinata* from principal component analyses.

In PCA for males, the total variation of the first two principal components was 63.2 %. In males on the two-dimensional plots of PC1 vs. PC2, the undescribed species could be distinctly separated from *M. sangzhiensis* and *M. spinata* (Fig. 3). The results of one-way ANOVA indicated that in males, the undescribed species was significantly different from *M. sangzhiensis* and *M. spinata* on many morphometric characters (all *p*-values < 0.05; Table 4). More detailed descriptions of results from morphological comparisons between the undescribed species and its congeners were presented in the following sections for describing the new species.

**Table 4.** Morphometric comparisons between *Megophrys qianbeiensis* sp. nov., *M. sangzhiensis*, and *M. spinata*. Units given in mm. Abbreviations for the species name: MQ, *Megophrys qianbeiensis* sp. nov.; MSZ, *M. sangzhiensis*; MSP, *M. spinata*. See abbreviations for morphometric characters in Materials and methods section.

	*Megophrys qianbeiensis* sp. nov.		*M. sangzhiensis*		*M. spinata*		*p*-value from ANOVA in male	
	males (N = 6)		males (N = 5)		males (N = 5)			
	Range	Mean ± SD	Range	Mean ± SD	Range	Mean ± SD	MQ vs. MSZ	MQ vs. MSP
SVL	49.3–58.2	54.3 ± 3.09	56.1–59.8	58.3 ± 1.56	51.2–56.2	53.9 ± 1.84	0.029	0.851
HDL	14.6–17.0	15.6 ± 0.93	16.1–18.0	17.2 ± 0.96	14.3–15.8	14.8 ± 0.59	0.027	0.124
HDW	18.3–21.0	19.9 ± 1.08	20.0–21.5	20.8 ± 0.55	18.4–19.1	18.7 ± 0.30	0.123	0.037
SL	6.7–7.2	6.9 ± 0.17	6.6–8.2	7.4 ± 0.58	5.7–7.1	6.4 ± 0.56	0.067	0.085
TYD	3.2–4.3	3.5 ± 0.39	3.1–3.7	3.4 ± 0.21	2.5–2.9	2.7 ± 0.15	0.639	0.001
IFE	7.8–10.9	9.7 ± 1.13	9.4–10.9	10.2 ± 0.54	8.5–9.6	9.1 ± 0.39	0.340	0.348
IAE	14.0–16.4	15.5 ± 0.86	16.1–17.3	16.8 ± 0.62	14.0–14.4	14.2 ± 0.18	0.019	0.009
NED	2.4–3.4	2.9 ± 0.36	3.0–3.9	3.4 ± 0.36	2.7–3.0	2.8 ± 0.11	0.060	0.618
NSD	3.5–4.3	3.8 ± 0.29	3.7–4.6	4.2 ± 0.34	3.4–4.1	3.8 ± 0.27	0.101	0.683
IND	5.7–7.5	6.7 ± 0.62	6.8–7.7	7.2 ± 0.34	5.8–6.3	6.0 ± 0.18	0.150	0.056
IOD	3.7–5.3	4.5 ± 0.59	4.7–5.8	5.1 ± 0.45	4.2–5.3	4.9 ± 0.44	0.117	0.257
ED	5.4–6.9	6.3 ± 0.58	6.1–7.4	6.7 ± 0.51	5.1–6.0	5.6 ± 0.34	0.280	0.041
UEW	5.1–6.2	5.6 ± 0.43	5.1–6.1	5.7 ± 0.42	4.5–6.1	5.0 ± 0.65	0.484	0.126
LAL	20.4–25.4	24.0 ± 1.84	26.2–28.2	26.9 ± 0.78	24.0–25.1	24.3 ± 0.44	0.014	0.654
LW	5.7–7.4	6.6 ± 0.55	6.1–6.7	6.4 ± 0.24	5.5–7.4	6.3 ± 0.72	0.394	0.364
HLL	76.9–93.7	87.8 ± 6.07	97.3–105.0	100.3 ± 2.84	85.7–99.0	91.8 ± 5.27	0.003	0.280
THL	24.1–28.4	26.6 ± 1.82	27.9–31.6	29.6 ± 1.59	26.1–29.8	27.6 ± 1.38	0.019	0.343
TL	25.0–32.4	29.2 ± 2.63	31.6–32.6	32.2 ± 0.42	28.9–30.4	29.6 ± 0.63	0.038	0.713
TW	7.0–8.7	8.0 ± 0.58	7.3–8.1	7.7 ± 0.32	6.0–8.0	7.0 ± 0.91	0.404	0.053
TFL	34.5–44.0	40.4 ± 3.58	42.9–46.1	44.2 ± 1.32	39.2–43.1	40.9 ± 1.55	0.062	0.761
FL	24.5–29.4	27.5 ± 1.95	26.7–29.9	28.9 ± 1.28	26.4–29.6	28.1 ± 1.16	0.201	0.531

### Bioacoustics comparisons

**Figure 4.**
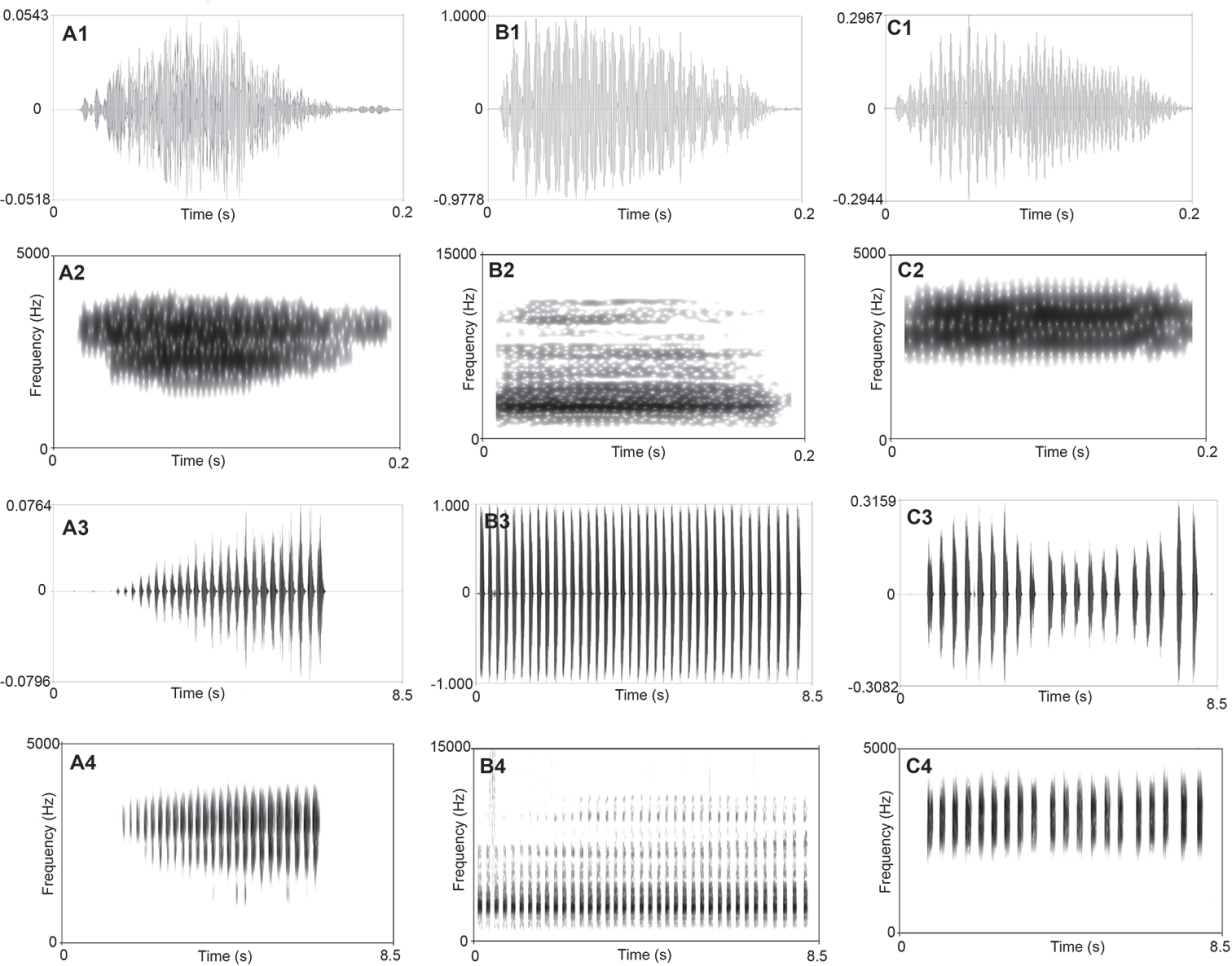
Visualization of advertisement calls of *Megophrys qianbeiensis* sp. nov., *M. sangzhiensis*, and *M. spinata***A1** waveform showing one note of *Megophrys qianbeiensis* sp. nov. **A2** sonogram showing one note of *Megophrys qianbeiensis* sp. nov. **A3** waveform showing 25 notes of one call of *Megophrys qianbeiensis* sp. nov. **A4** sonogram showing 25 notes of one call of *Megophrys qianbeiensis* sp. nov. **B1** waveform showing one note of *M. sangzhiensis***B2** sonogram showing one note of *M. sangzhiensis***B3** waveform showing 38 notes of one call of *M. sangzhiensis*. **B4** sonogram showing 38 notes of one call of *M. sangzhiensis***C1** waveform showing one note of *M. spinata***C2** sonogram showing one note of *M. spinata***C3** waveform showing 20 notes of one call of *M. spinata***C4** sonogram showing 20 notes of one call of *M. spinata*.

There were many differences in sonograms and waveforms of calls between the undescribed species, *M. sangzhiensis*, and *M. spinata* (Fig. 4; Table 5). Firstly, in the note interval, the undescribed species were shorter than those of both *M. sangzhiensis* and *M. spinata*. Secondly, the dominant frequency of call in the undescribed species was lower than both of *M. sangzhiensis* and *M. spinata*. Thirdly, the amplitude of the undescribed species beginning with moderately high energy pulses, increasing slightly to a maximum by approximately mid note, and then decreasing towards the end of each note, in *M. sangzhiensis* beginning with maximum energy pulses and then decreasing towards the end of note, and in *M. spinata* beginning with lower energy pulses, then increasing to the maximum by approximately one-four note and then decreasing to the mid note then increasing to the second highest energy pulses and then decreasing towards the end of note.

**Table 5.** Comparisons of characteristics of advertisement calls of *Megophrys qianbeiensis* sp. nov., *M. sangzhiensis*, and *M. spinata*.

Call character	*Megophrys qianbeiensis* sp. nov.		*M. sangzhiensis*		*M. spinata*	
	Range	Mean ± SD	Range	Mean ± SD	Range	Mean ± SD
Number of notes in a call	14–26	22.5 ± 4.4	38	/	7–28	17 ± 7.92
Call duration (ms)	2832–5621	4413 ± 972	8152	/	1500–6623	3905 ± 2010
Call interval (ms)	6812–14387	10878 ± 2701	/	/	592–5770	2708 ± 1863.33
Note duration (ms)	129– 211	167 ± 0.02	107–155	120.3 ± 8.73	107–123	114 ± 3.79
Note interval (ms)	34–94	57 ± 0.01	72–132	95.6 ± 13.89	113–232	147 ± 33.12
Dominant frequency (Hz)	2250–3000	2469 ± 197.47	10380–13200	11795 ± 670.58	4260–4589	4416 ± 130.04
Temperature (°C)	20.5		18.5		19.0	

### Taxonomic accounts

#### Megophrys qianbeiensis sp. nov.

Taxon classificationAnimaliaAnuraMegophrys

9443A950-3802-5A20-8423-9431B10C1C33

http://zoobank.org/C6C89A51-8178-4C7B-A100-80C0D2D42AD3

Figures 4A1–C1 , 5 , 6A1–A6 , 7 ; Tables 1 , 2 , 4 , 5 , Suppl. material 2: Table S2

##### Type material

**Figure 5.**
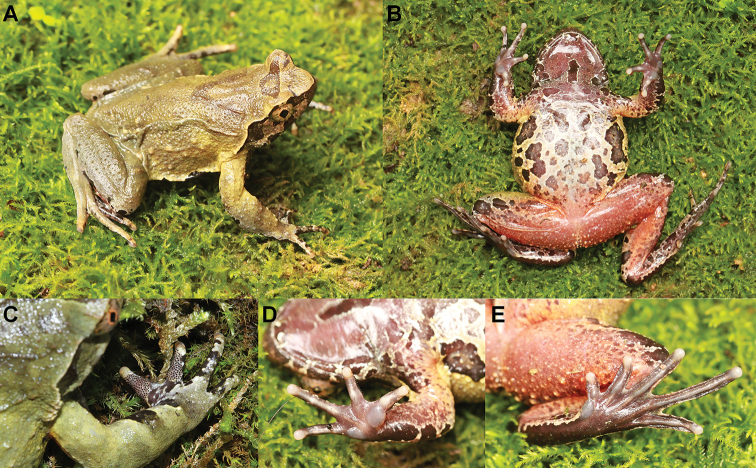
Photos of the holotype CIBTZ20190608017 of *Megophrys qianbeiensis* sp. nov. in life **A** dorsal view **B** ventral view **C** dorsal view of hand **D** ventral view of hand **E** ventral view of foot.

**Figure 6.**
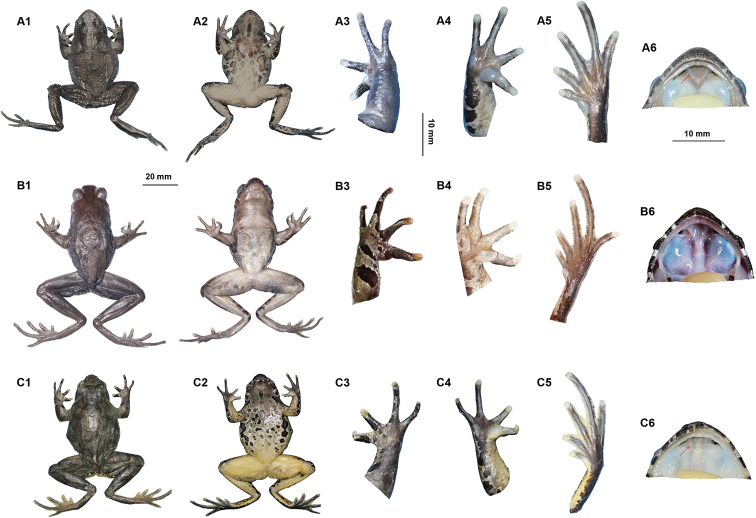
Photos of specimens of *Megophrys qianbeiensis* sp. nov., *M. sangzhiensis* and *M. spinata***A1–A6** dorsal view, ventral view, dorsal view of hand, ventral view of hand, ventral view of foot and view of oral cavity of the holotype specimen CIBTZ20190608017 of *Megophrys qianbeiensis* sp. nov. **B1–B6** dorsal view, ventral view, dorsal view of hand, ventral view of hand, ventral view of foot and view of oral cavity of CIBSZ2012062005 of *M. sangzhiensis***C1–C6** dorsal view, ventral view, dorsal view of hand, ventral view of hand, ventral view of foot and view of oral cavity of CIBLS20190801001 of *M. spinata*. Arrow point to vomerine ridge.

**. *Holotype*.** CIBTZ20190608017 (Figs 5, 6), adult male, from Huanglian Nature Reserve, Tongzi County, Guizhou Province, China (28.498056°N, 107.046944°E, ca. 1500 m a.s.l.), collected by Shi-Ze Li 8 June 2019.

***Paratype*.** Four adult males from the same place as holotype, and one from Kuankuoshui National Nature Reserve (28.21835°N, 107.166388°E, ca.1520 m a.s.l.) collected by Shi-Ze Li. CIBKKS20180722001 collected 22 July 2018 from Kuankuoshui National Nature Reserve and CIBTZ20160715003 collected 15 July 2016, CIBTZ20190608015, CIBTZ20190608016 and CIBTZ20190608018 collected 8 June 2019 from Kuankuoshui National Nature Reserve.

##### Diagnosis.

*Megophrys qianbeiensis* sp. nov. is assigned to the genus *Megophrys* based on molecular phylogenetic analyses and the following generic diagnostic characters: snout shield-like; projecting beyond the lower jaw; canthus rostralis distinct; chest glands small and round, closer to the axilla than to midventral line; femoral glands on rear part of thigh; vertical pupils.

*Megophrys qianbeiensis* sp. nov. could be distinguished from its congeners by a combination of the following morphological characters: body size moderate (SVL 49.3–58.2 mm in males); vomerine ridges present distinctly, vomerine teeth present; tongue feebly notched behind; tympanum distinctly visible, oval; two metacarpal tubercles in hand; toes with one-third webbing and wide lateral fringes; heels overlapped when thighs are positioned at right angles to the body; tibiotarsal articulation reaching the level between tympanum and eye when leg stretched forward; an internal single subgular vocal sac present in male; in breeding male, the nuptial pads with large and sparse black nuptial spines present on the dorsal bases of the first two fingers.

##### Description of holotype.

(Figs 5, 6). SVL 56.3 mm; head width larger than head length (HDW/HDL ratio ca. 1.3); snout obtusely pointed, protruding well beyond the margin of the lower jaw in ventral view; loreal region vertical and concave; canthus rostralis well-developed; top of head flat in dorsal view; eye large, eye diameter 44.5 % of head length; pupils vertical; nostril orientated laterally, closer to snout than eye; tympanum distinct, TYP/EYE ratio 0.49; vomerine ridges present distinctly as V-shape, vomerine teeth present; margin of tongue smooth, feebly notched behind.

Forelimbs slender, the length of lower arm and hand 42.6 % of SVL; fingers burly, relative finger lengths: II < I < V < III; tips of digits globular, without lateral fringes; subarticular tubercle distinct at the base of each finger; two metacarpal tubercles, prominent, oval-shaped, the inner one bigger than the outer one.

Hindlimbs slender, 1.54 times of SVL; heels overlapping when thighs are positioned at right angles to the body, tibiotarsal articulation reaching tympanum to eye when leg stretched forward; tibia length longer than thigh length; relative toe lengths I < II < V < III < IV; tips of toes round, slightly dilated; subarticular tubercles present on the base of each toes ; toes with one-third webbing and relative wide lateral fringe; inner metatarsal tubercle oval-shaped; outer metatarsal tubercle absent.

Dorsal skin rough, with numerous granules with black spins; several large warts scattered on flanks; tubercles on the dorsum forming a weak V-shaped ridge; two discontinuous dorsolateral parallel ridges on either side of the V-shaped ridges; an inverted triangular brown speckle between two upper eyelids; several tubercles on the flanks and dorsal surface of thighs and tibias; supratympanic fold distinct.

Ventral surface smooth with numerous white granules; glands on chest indistinct; femoral glands on rear of thighs, numerous white granules on outer thighs; posterior end of the body distinctly protruding and forming an arc-shaped swelling above the anal region.

##### Coloration of holotype in life

(Fig. 5). An inverted triangular brown speckle between the eyes; V-shaped ridges on the dorsum with brown speckle, on transverse bands on the dorsal surface of the thigh and shank; several dark brown and white vertical bars on the lower and upper lip; belly whitish grey with dark brown marbling; ventral surface of posterior limb orange with numerous granules; palms, soles and tip of digits uniform purple grey; femoral glands white.

##### Coloration of holotype in preservation

(Fig. 6). Color of dorsal surface fades to brownness; the inverted triangular brown speckle between the eyes and V-shaped ridges on dorsum indistinct; ventral surface greyish white; creamy-white substitutes the purple grey on tip of digits; the posterior of ventral surface of body, inner of thigh and upper of tibia fades to creamy-white.

##### Variation.

**Figure 7.**
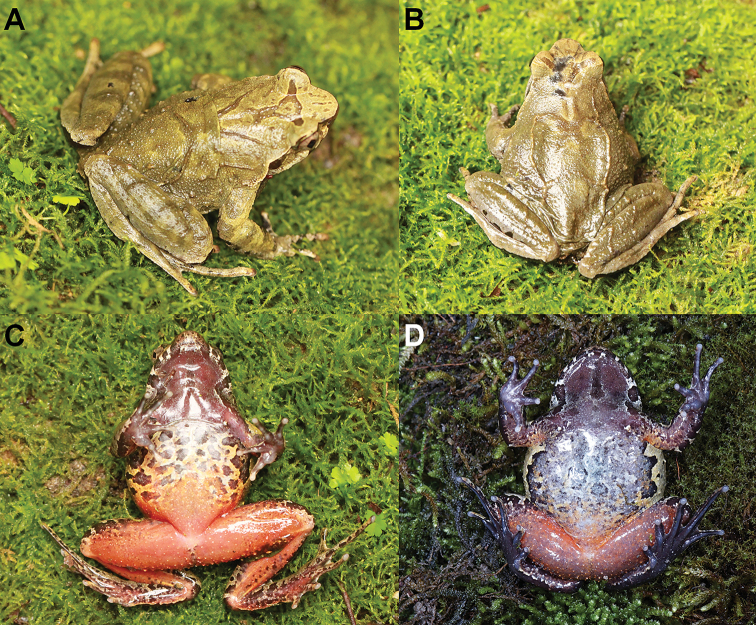
Color variation in *Megophrys qianbeiensis* sp. nov. **A** dorsolateral view of the specimen CIBTZ20160715003 **B** dorsolateral view of the specimen CIBTZ20190608015 **C** ventral view of the male specimen CIBTZ20190608015 **D** ventral view of the specimen CIBKKS20180722001.

In CIBTZ20160715003 the dorsolateral parallel ridges are short, just a little bit above the shoulder (Fig. 7A); in CIBTZ20190608015 the X-shaped marking on back of trunk consists of a ridge with brown spots (Fig. 7B) and the posterior belly are orange with black spots on the flank belly (Fig. 7C); in CIBKKS20180722001 the belly is grey brown with some white spots (Fig. 7D).

##### Advertisement call.

The call description is based on recordings of the holotype CIBTZ20190608017 (Fig. 4) from the shrub leaf near the streamlet, and the ambient air temperature was 20.5 °C. Each call consists of 14–26 (mean 22.5 ± 4.4, *N* = 6) notes. Call duration was 2832–5621 ms (mean 4413 ± 972, *N* = 6). Call interval was 6812–14387 ms (mean 10878 ± 2701, *N* = 5). Each note had a duration of 129–211 ms (mean 167 ± 0.02, *N* = 135) and the intervals between notes 34–94 ms (mean 57 ± 0.01, *N* = 128). Amplitude modulation within note was apparent, beginning with moderately high energy pulses, increasing slightly to a maximum by approximately mid note, and then decreasing towards the end of each note. The average dominant frequency was 2469 ± 197.47 (2250–3000 Hz, *N* = 6).

##### Secondary sexual characters.

Adult males have a single subgular vocal sac. In breeding males, brownish red nuptial pads are present on the dorsal bases of the first two fingers with big and sparse black nuptial spines (Fig. 5A).

##### Comparisons.

By having moderate body size (minimum SVL > 49.8 mm in males), *Megophrys qianbeiensis* sp. nov. differs from *M. aceras*, *M. acuta*, *M. angka*, *M. ancrae*, *M. baluensis*, *M. baolongensis*, *M. binchuanensis*, *M. binlingensis*, *M. boettgeri*, *M. brachykolos*, *M. caobangensis*, *M. cheni*, *M. daweimontis*, *M. dongguanensis*, *M. dringi*, *M. edwardinae*, *M. elfina*, *M. fansipanensis*, *M. feii*, *M. gerti*, *M. hansi*, *M. hoanglienensis*, *M. huangshanensis*, *M. insularis*, *M. jiangi*, *M. jinggangensis*, *M. jiulianensis*, *M. kuatunensis*, *M. lancip*, *M. leishanensis*, *M. lini*, *M. lishuiensis*, *M. longipes*, *M. major*, *M. microstoma*, *M. minor*, *M. monticola*, *M. mufumontana*, *M. nankunensis*, *M. nanlingensis*, *M. obesa*, *M. ombrophila*, *M. oropedion*, *M. pachyproctus*, *M. palpebralespinosa*, *M. parallela*, *M. parva*, *M. rubrimera*, *M. serchhipii*, *M. shimentaina*, *M. shunhuangensis*, *M. tuberogranulata*, *M. vegrandis*, *M. wawuensis*, *M. wugongensis*, *M. wuliangshanensis*, *M. wushanensis*, *M. xianjuensis*, *M. zhangi*, *M. zunhebotoensis*, *M. xiangnanensis*, and *M. yangmingensis* (vs. minimum SVL < 48.0 mm).

By having moderate body size (minimum SVL < 59.0 mm in males), *Megophrys qianbeiensis* sp. nov. differs from *M. auralensis*, *M. carinense*, *M. caudoprocta*, *M. caudoprocta*, *M. chuannanensis*, *M. feae*, *M. gigantica*, *M. glandulosa*, *M. himalayana*, *M. kalimantanensis*, *M. kobayashii*, *M. ligayae*, *M. mangshanensis*, *M. orientalis*, *M. periosa*, *M. platyparietus*, *M. popei*, *M. shapingensis*, and *M. shuichengensis* (vs. minimum SVL > 60.0 mm).

By having vomerine teeth, *Megophrys qianbeiensis* sp. nov. differs from *M. aceras*, *M. acuta*, *M. angka*, *M. auralensis*, *M. baolongensis*, *M. binchuanensis*, *M. binlingensis*, *M. boettgeri*, *M. brachykolos*, *M. caobangensis*, *M. cheni*, *M. chishuiensis*, *M. dringi*, *M. jiangi* , *M. leishanensis*, *M. lini* , *M. lishuiensis*, *M. major* , *M. microstoma*, *M. minor*,. *M. mirabilis*, *M. mufumontana*, *M. nankiangensis*, *M. obesa*, *M. ombrophila*, *M. shapingensis*, *M. shuichengensis*, *M. shunhuangensis*, *M. tuberogranulata*, *M. vegrandis*, *M. wawuensis*, *M. wugongensis*, *M. wuliangshanensis*, *M. wushanensis*, *M. xianjuensis*, *M. xiangnanensis*, and *M. yangmingensis* (vs. absent).

By the absence of horn-like tubercle at the edge of each upper eyelid, *Megophrys qianbeiensis* sp. nov. differs from *M. aceras*, *M. acuta*, *M. angka*, *M. ancrae*, *M. auralensis*, *M. baluensis*, *M. baolongensis*, *M. boettgeri*, *M. brachykolos*, *M. caobangensis*, *M. carinense*, *M. caudoprocta*, *M. cheni*, *M. chishuiensis*, *M. chuannanensis*, *M. daweimontis*, *M. dongguanensis*, *M. dringi*, *M. edwardinae*, *M. elfina*, *M. fansipanensis*, *M. feae*, *M. feii*, *M. flavipunctata*, *M. gerti*, *M. glandulosa*, *M. hansi*, *M. himalayana*, *M. hoanglienensis*, *M. huangshanensis*, *M. insularis*, *M. intermedia*, *M. jiangi*, *M. jingdongensis*, *M. jinggangensis*, *M. jiulianensis*, *M. kalimantanensis*, *M. koui*, *M. kuatunensis*, *M. lancip*, *M. leishanensis*, *M. lekaguli*, *M. liboensis*, *M. ligayae*, *M. lini*, *M. lishuiensis*, *M. longipes*, *M. mangshanensis*, *M. medogensis*, *M. microstoma*, *M. mirabilis*, *M. montana*, *M. mufumontana*, *M. nankunensis*, *M. nanlingensis*, *M. nasuta*, *M. obesa*, *M. ombrophila*, *M. omeimontis*, *M. oreocrypta*, *M. orientalis*, *M. palpebralespinosa*, *M. parallela*, *M. parva*, *M. periosa*, *M. platyparietus*, *M. popei*, *M. rubrimera*, *M. shimentaina*, *M. shuichengensis*, *M. shunhuangensis*, *M. stejnegeri*, *M. synoria*, *M. vegrandis*, *M. wugongensis*, *M. xianjuensis*, *M. xiangnanensis*, and *M. yangmingensis* (vs. present).

With the tongue feebly notched behind, *Megophrys qianbeiensis* sp. nov. differs from *M. aceras*, *M. acuta*, *M. angka*, *M. auralensis*, *M. brachykolos*, *M. caobangensis*, *M. caudoprocta*, *M. dongguanensis*, *M. elfina*, *M. hansi*, *M. jiangi*, *M. jinggangensis*, *M. lancip*, *M. leishanensis*, *M. lekaguli*, *M. lini*, *M. lishuiensis*, *M. megacephala*, *M. mufumontana*, *M. nankunensis*, *M. obesa*, *M. ombrophila*, *M. orientalis*, *M. palpebralespinosa*, *M. parallela*, *M. parva*, *M. shunhuangensis*, *M. takensis*, *M. wushanensis*, and *M. xianjuensis* (vs. tongue not notched behind in the latter), and differs from *M. cheni*, *M. damrei*, *M. dringi*, *M. flavipunctata*, *M. gigantica*, and *M. popei* (vs. tongue notched behind).

By having lateral wide fringes on toes, *Megophrys qianbeiensis* sp. nov. differs from *M. angka*, *M. baolongensis*, *M. brachykolos*, *M. caobangensis*, *M. damrei*, *M. daweimontis*, *M. dongguanensis*, *M. fansipanensis*, *M. feae*, *M. himalayana*, *M. hoanglienensis*, *M. huangshanensis*, *M. insularis*, *M. jiangi*, *M. jiulianensis*, *M. kalimantanensis*, *M. koui*, *M. leishanensis*, *M. lekaguli*, *M. lishuiensis*, *M. major*, *M. mangshanensis*, *M. medogensis*, *M. megacephala*, *M. microstoma*, *M. minor*, *M. nankunensis*, *M. obesa*, *M. ombrophila*, *M. oreocrypta*, *M. oropedion*, *M. pachyproctus*, *M. parva*, *M. periosa*, *M. shunhuangensis*, *M. takensis*, *M. tuberogranulata*, *M. wawuensis*, *M. wugongensis*, *M. wuliangshanensis*, and *M. xianjuensis* (vs. lacking lateral fringes on toes).

By toes with one-third webs, *Megophrys qianbeiensis* sp. nov. differs from *M. aceras*, *M. acuta*, *M. angka*, *M. auralensis*, *M. baluensis*, *M. baolongensis*, *M. binchuanensis*, *M. binlingensis*, *M. boettgeri*, *M. brachykolos*, *M. caobangensis*, *M. caudoprocta*, *M. cheni*, *M. chuannanensis*, *M. damrei*, *M. daweimontis*, *M. dongguanensis*, *M. dringi*, *M. elfina*, *M. fansipanensis*, *M. feae*, *M. feii*, *M. flavipunctata*, *M. gerti*, *M. gigantica*, *M. glandulosa*, *M. hansi*, *M. hoanglienensis*, *M. huangshanensis*, *M. insularis*, *M. jiangi*, *M. jinggangensis*, *M. jiulianensis*, *M. kalimantanensis*, *M. koui*, *M. kuatunensis*, *M. lancip*, *M. leishanensis*, *M. lekaguli*, *M. liboensis*, *M. lini*, *M. lishuiensis*, *M. longipes*, *M. major*, *M. mangshanensis*, *M. medogensis*, *M. medogensis*, *M. megacephala*, *M. microstoma*, *M. minor*, *M. mufumontana*, *M. nankiangensis*, *M. nankunensis*, *M. nanlingensis*, *M. obesa*, *M. ombrophila*, *M. omeimontis*, *M. oropedion*, *M. pachyproctus*, *M. parva*, *M. periosa*, *M. robusta*, *M. rubrimera*, *M. serchhipii*, *M. shunhuangensis*, *M. takensis*, *M. tuberogranulata*, *M. vegrandis*, *M. wawuensis*, *M. wugongensis*, *M. wuliangshanensis*, *M. wushanensis*, *M. xianjuensis*, and *M. zhangi* (vs. with rudimentary or without webs).

By heels overlapping when thighs are positioned at right angles to the body, *Megophrys qianbeiensis* sp. nov. differs from *M. acuta*, *M. brachykolos*, *M. dongguanensis*, *M. huangshanensis*, *M. kuatunensis*, *M. nankunensis*, *M. obesa*, *M. ombrophila*, and *M. wugongensis* (vs. not meeting).

By the tibiotarsal articulation reaching to the level between tympanum and eye when leg stretched forward, *Megophrys qianbeiensis* sp. nov. differs from *M. daweimontis*, *M. glandulosa*, *M. lini*, *M. major*, *M. medogensis*, and *M. obesa* (vs. reaching the anterior corner of the eye or beyond eye or nostril and tip of snout).

By having an internal single subgular vocal sac in male, *Megophrys qianbeiensis* sp. nov. differs from *M. caudoprocta*, *M. shapingensis*, and *M. shuichengensis* (vs. vocal sac absent).

The congeners *M. carinense* and *M. jiangi* have sympatric distribution with *Megophrys qianbeiensis* sp. nov. (Fei et al. 2012). The new species can be distinguished from these species by a series of morphological characters as follows. The new species differs from *M. carinense* in the smaller body size in the new species (adult males with 49.3–58.2 mm vs. adult males with 92–123 mm in the latter), a horn-like tubercle at the edge of each upper eyelid absent (vs. prominent in the latter), the tongue feebly notched behind (vs. notched behind in the latter). The new species differs from *M. jiangi* by a larger body size (49.3–58.2 mm in males in the new species vs. 34.4–39.2 mm in the latter), a horn-like tubercle at the edge of each upper eyelid absent (vs. present in the latter), the tongue feebly notched behind (vs. notched behind in the latter), presence of wide lateral fringes on the toes (vs. lacking in the latter), and toes with one-third webbing (vs. rudimentary webbing in the latter).

*Megophrys qianbeiensis* sp. nov. is phylogenetically closest to *M. sangzhiensis* and *M. spinata*. The new species differs from *M. sangzhiensis* by the following characters: horn-like tubercle absent at the edge of each upper eyelid (vs. present in the latter), toes with one-third webs (vs. with rudimentary webbing in the latter), vomerine ridges present distinctly as V-shape and vomerine teeth present (vs. vomerine ridges separated and weak, vomerine teeth absent in the latter), tibiotarsal articulation reaching to the level between tympanum and eye when leg stretched forward (vs. reaching the anterior corner of eye in the latter), spines on nuptial pads on the first two fingers larger and sparser (vs. finer and thicker in the latter), and having significantly higher ratios of HDL, LAL, HLL, TL, and IAE to SVL. On bioacoustics, the new species differs from *M. sangzhiensis* in the following characters: lower dominant frequency (2250–3000 Hz in the new species vs. 10380 – 13200 Hz in the latter), the amplitude beginning with moderately high energy pulses, increasing slightly to a maximum by approximately mid note, and then decreasing towards the end of each note (vs. beginning with maximum energy pulses and then decreasing towards the end of note in the latter).

The new species differs from *M. spinata* by the following characters: tibiotarsal articulation reaching the level between tympanum to eye when leg stretched forward (vs. reaching the anterior corner of eye in the latter), present distinctly as V-shape and vomerine teeth present (vs. vomerine ridges separated and weak, vomerine teeth absent in the latter), spines on nuptial pads on the first two fingers little weaker (vs. spines larger in the latter), and having significantly higher ratios of HDW, ED, LAL, TYD and IAE to SVL. On bioacoustics, the new species differs from *M. spinata* in the following characters: lower dominant frequency (2250–3000 Hz in the new species vs. 4260–4589 Hz in the latter), the amplitude beginning with moderately high energy pulses, increasing slightly to a maximum by approximately mid note, and then decreasing towards the end of each note (vs. beginning with lower energy pulses, then increasing to the maximum by approximately one-four note and then decreasing to the mid note then increasing to the second highest energy pulses and then decreasing towards the end of note in the latter).

##### Distribution and habitats.

**Figure 8.**
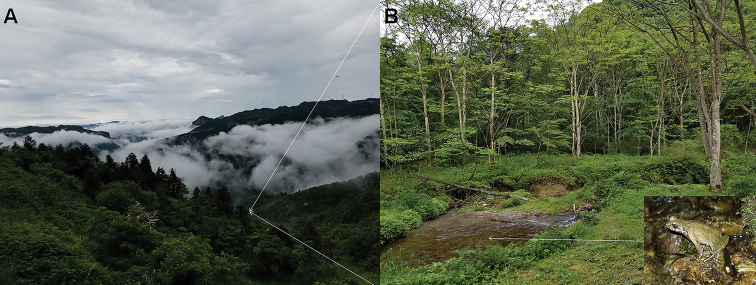
Habitats of *Megophrys qianbeiensis* sp. nov. in the type locality, Huanglian Nature Reserve, Tongzi County, Guizhou Province, China **A** landscape of montane forests in the type locality **B** a mountain stream where toads of the new species live (*insert* the holotype CIBTZ20190608017standing on the stone).

*Megophrys qianbeiensis* sp. nov. is known from Huanglian Nature Reserve, Tongzi County and Kuankuoshui National Nature Reserve, Suiyang County, Guizhou Province, China at elevations between 1400–1600 m. The individuals of the new species were frequently found on stone in the streams surrounded by evergreen broadleaved forests (Fig. 8), and three sympatric amphibian species were found, i.e., *Megophrys jiangi*, *Odorrana margaratae* (Liu, 1950), and *Zhangixalus omeimontis* (Stejneger, 1924).

##### Etymology.

The specific epithet *qianbeiensis* refers to northern part of Guizhou, also called “黔”, the province where the type locality of the species belongs to. We propose the common English name “Qianbei horned toad” and Chinese name “Qian Bei Jiao Chan (黔北角蟾)”.

## Discussion

The new species *Megophrys qianbeiensis* sp. nov. resembles *M. spinata* and *M. sangzhiensis*, and detailed comparisons with different data are important for recognizing them. Our molecular phylogenetic data on mitochondrial DNA and morphological comparisons both separated the new species from the two closely related species. *Megophrys spinata* were reported to be distributed widely through the provinces of Sichuan, Guizhou, Hunan, Chongqing, Yunnan, and Guangxi (Fei et al. 2012), but detailed investigations with multiple data suggested that several populations of the species should contain cryptic species (including *Megophrys qianbeiensis* sp. nov. and *M. sangzhiensis*). In recent years, many new species of the genus *Megophrys* have been gradually described, of which a large part of number was found in China (Frost 2020). To date, among the 106 species of *Megophrys*, 56 species were discovered in China. Even so, many cryptic species still need to be described just in southern China (Chen et al. 2017; Liu et al. 2018).

South-western China has long been proposed as biodiversity hotspot (Myers et al. 2000). Guizhou Province is an important part of southwest China, especially with the particular environments of karst rocky desertifcation, and knowledge of biodiversity levels and/or patterns are still seriously lacking. Recently, a series of new amphibian species were described from this province (Zhang et al. 2017; Li et al. 2018a, b, 2019a, b; Lyu et al. 2019; Wang et al. 2019c; Luo et al. 2020; Liu et al. 2020; Wei et al. 2020; Xu et al. 2020), indicating that species diversity of amphibians in this region is highly underestimated. It is urgent for herpetologists to conduct comprehensive and in-depth surveys to explore the level of amphibian species diversity in this region under accelerating climate changes. Obviously, more work should be conducted on detecting the differentiation of the populations and further describe the cryptic species in this region.

## Supplementary Material

XML Treatment for Megophrys qianbeiensis

## Supplementary materials

Supplementary material 1 Table S1 Data type molecular data Explanation note Uncorrected p-distances between the *Megophrys* species based on COI gene sequences https://binary.pensoft.net/file/460608Haijun Su, Shengchao Shi, Yanqing Wu, Guangrong Li, Xiaogang Yao, Bin Wang, Shize Li

Supplementary material 2 Table S2 Data type morphological data Explanation note Diagnostic characters separating the new species described in this study from other species of *Megophrys* https://binary.pensoft.net/file/460609Bin Wang
