# Supplementary material for: Description of a new horned toad of Megophrys Kuhl & Van Hasselt, 1822 (Anura, Megophryidae) from southwest China
Source: Zookeys. 2020 Oct 7;974:131–59. doi: 10.3897/zookeys.974.56070 (PMC7557532; doi:10.3897/zookeys.974.56070)
Supplement: Supplementary material 2 — Table S2 [file zookeys-974-131-s002.docx]

**Table S2**. Diagnostic characters separating the new species described in this study from other species of *Megophrys*.

| **Species** | **SVL** | | **Horn-like tubercle at edge of upper eyelidlong. point (+++); slightly large (++), small (+), absent or indistinct (‒)** | **Vomerine teeth. present (+), or absent (‒)** | **Tongue. notched (++), feebly notched (+), or not notched (‒)** | **Lateral fringes on toes. wide (++), narrow (+), lacking (‒)** | **Toes. at least one-fourth webbed (+++), at most one-fourth webbed (++), with rudimentary webbing (+), or without webbing (‒)** |
| --- | --- | --- | --- | --- | --- | --- | --- |
|  | **Males** | **Females** |  |  |  |  |  |
| *Megophrys qianbeiensis* sp. nov. | 49.3–58.2 | / | - | + | + | ++ | +++ |
| *Megophrys sangzhiensis* | 54.7 | / | + | - | + | + | + |
| *Megophrys spinata* | 47.2–54.4 | 54.0–55.0 | - | - | + | ++ | +++ |
| *Megophrys aceras* | 27.1–33.0 | 28.1–33.6 | ++ | - | - | + | + |
| *Megophrys acuta* | 27.1–33.0 | 28.1–33.6 | ++ | - | - | + | + |
| *Megophrys angka* | 31.2–32.1 | 37.5–39.2 | + | - | - | - | + |
| *Megophrys ancrae* | 39.1–45.0 | 48.9 | + | + | +or- | + | +or++ |
| *Megophrys auralensis* | 76.7 | / | + | - | - | + | + |
| *Megophrys baluensis* | 41–45 | 54–70 | + | + | / | + | + |
| *Megophrys baolongensis* | 42.0–45.0 | / | + | - | + | - | - |
| *Megophrys binchuanensis* | 32.0–36.0 | 40.2–42.5 | - | - | +or- | ++ | + |
| *Megophrys binlingensis* | 45.1–51.0 | / | - | - | + | / | + |
| *Megophrys boettgeri* | 34.5–37.8 | 39.7–46.8 | + | - | + | ++ | + |
| *Megophrys brachykolos* | 33.7–39.3 | 33.9–45.9 | + | - | - | - | + |
| *Megophrys caobangensis* | 34.9–38.9 | / | + | - | - | - | + |
| *Megophrys carinense* | 92–123 | 137 | ++ | + | + | ++ | ++ |
| *Megophrys caudoprocta* | 81.3 | / | ++ | + | - | / | + |
| *Megophrys cheni* | 26.2–29.5 | 31.8–34.1 | + | - | ++ | ++ | + |
| *Megophrys chishuiensis* | 43.4–44.1 | 44.8–49.8 | + | - | - | - | - |
| *Megophrys chuannanensis* | 91–109 | / | ++ | + | / | ++ | + |
| *Megophrys damrei* | 57.1 | 69.1 | - | + | ++ | - | + |
| *Megophrys daweimontis* | 34.0–37.0 | 40.0–46.0 | + | + | / | - | - |
| *Megophrys dongguanensis* | 30.2–39.3 | / | + | + | - | - | + |
| *Megophrys dringi* | 26.2–29.5 | 31.8–34.1 | + | - | ++ | ++ | + |
| *Megophrys edwardinae* | 39–42 | 69–82 | + | - | / | / | / |
| *Megophrys elfina* | 26.9–33.9 | 35.1–36.5 | + | - | - | + | + |
| *Megophrys fansipanensis* | 30.9–44.3 | 41.7–42.5 | + | + | + | - | - |
| *Megophrys feae* | 78–102 | 91–111.4 | ++ | + | / | - | + |
| *Megophrys feii* | 24.3–25.1 | 28.2–28.9 | + | - | + | ++ | + |
| *Megophrys flavipunctata* | 56.9–68.4 | 68.0–74.6 | + | + | ++ | + | + |
| *Megophrys gerti* | 32–34.8 | 41.4–45.8 | ++ | - | / | / | - |
| *Megophrys gigantica* | 80.5–107.0 | 110.4–115.4 | - | - | ++ | ++ | + |
| *Megophrys glandulosa* | 76.0–81.0 | 77.0–100.0 | + | + | + | ++ | + |
| *Megophrys hansi* | 35.3–43 | 53.5 | ++ | - | - | / | - |
| *Megophrys himalayana* | 68.0–73.5 | 83.9 | + | + | / | - | ++ |
| *Megophrys hoanglienensis* | 37.4–47.6 | 59.6 | + | + | + | - | - |
| *Megophrys huangshanensis* | 36.0–41.6 | 44.2 | + | - | + | - | - |
| *Megophrys insularis* | 36.8–41.2 | 47.1 | + | + | + | - | + |
| *Megophrys intermedia* | / | / | ++ | + | / | ++ | / |
| *Megophrys jiangi* | 34.4–39.2 | 39.5–40.4 | + | - | - | - | - |
| *Megophrys jingdongensis* | 53.0–56.5 | 63.5 | + | + | + | ++ | +++ |
| *Megophrys jinggangensis* | 35.1–36.7 | 38.4–41.6 | ++ | + | - | + | + |
| *Megophrys jiulianensis* | 30.4–33.9 | 34.1–37.5 | + | + | + | - | + |
| *Megophrys kalimantanensis* | 64.8–101.1 | 109.6–116.4 | ++ | + | + | - | + |
| *Megophrys kobayashii* | 99 | 109 | / | + | / | / | / |
| *Megophrys koui* | / | / | ++ | / | / | - | - |
| *Megophrys kuatunensis* | 26.2–29.6 | 37.4 | + | - | + | + | - |
| *Megophrys lancip* | 37.9–47.7 | 38.7–82.5 | ++ | + | - | / | + |
| *Megophrys leishanensis* | 30.4–38.7 | 42.3 | + | - | - | - | + |
| *Megophrys lekaguli* | 55.6–66.6 | 71.8–94.0 | + | + | - | - | + |
| *Megophrys liboensis* | 34.7–67.7 | 60.8–70.6 | +++ | + | + | ++ | + |
| *Megophrys ligayae* | 60 | 90 | + | + | / | / | / |
| *Megophrys lini* | 34.1–39.7 | 37.0–39.9 | + | - | - | ++ | + |
| *Megophrys lishuiensis* | 30.7–34.7 | 36.9–40.4 | + | - | - | - | - |
| *Megophrys longipes* | 47.0 | 65.0 | + | + | + | / | + |
| *Megophrys major* | 34.5–41.2 | / | - | - | + | - | + |
| *Megophrys mangshanensis* | 62.5 | 73 | + | + | + | - | - |
| *Megophrys maosonensis* | / | / | / | + | + | + | / |
| *Megophrys medogensis* | 57.2–68.0 | / | + | + | + | - | +or- |
| *Megophrys megacephala* | 45.9–53.4 | 64.4 | - | + | - | - | + |
| *Megophrys microstoma* | 28–36 | 47–49 | ++ | - | / | - | - |
| *Megophrys minor* | 32.2–40.5 | 42.0–48.2 | - | - | + | - | + |
| *Megophrys mirabilis* | 55.8–61.4 | 68.5–74.8 |  | - | - | + | + |
| *Megophrys montana* | 38.1–53.9 | 45.7–99.5 | ++ | + | / | / | / |
| *Megophrys monticola* | / | 40.5 | / | / | / | / | / |
| *Megophrys mufumontana* | 30.1–30.8 | 36.3 | + | - | - | + | + |
| *Megophrys nankiangensis* | / | 44.0–52.9 | - | - | + | + | + |
| *Megophrys nankunensis* | 29.9–34.9 | 39.4–41.9 | + | + | - | - | + |
| *Megophrys nanlingensis* | 30.5–37.3 | / | + | + | + | + | + |
| *Megophrys nasuta* | 69.3–97.5 | 45.9–134.6 | ++ | + | / | / | / |
| *Megophrys obesa* | 35.6 | 37.5–41.2 | + | - | - | - | + |
| *Megophrys ombrophila* | 27.4–34.5 | 32.8–35.0 | + | - | - | - | - |
| *Megophrys omeimontis* | 56.0–59.5 | 68.0–72.5 | + | + | + | + | + |
| *Megophrys oreocrypta* | / | 94.9 | + | + | / | - | ++ |
| *Megophrys orientalis* | 76.8–82.7 | 88.6 | ++ | + | - | ++ | ++ |
| *Megophrys oropedion* | 32.8–39.2 | 44.1-48.7 | - | + | + | - | - |
| *Megophrys pachyproctus* | 35.3–36.2 | 35.8 | - | + | + | - | - |
| *Megophrys palpebralespinosa* | 36.2–38.0 | / | ++ | + | - | ++ | +++ |
| *Megophrys parallela* | 37.7–46.3 | 35.5–58.3 | + | + | - | / | / |
| *Megophrys parva* | 37.0–44.0 | 45.0–54.0 | + | + | - | - | +or- |
| *Megophrys periosa* | 71.3–93.8 | 112 | + | + | / | - | + |
| *Megophrys platyparietus* | 88.5–113.0 | 118.5–131.0 | ++ | + | + | ++ | ++ |
| *Megophrys popei* | 70.7–83.5 | 86.2 | ++ | + | ++ | + | +++ |
| *Megophrys robusta* | / | 114.0 | / | + | + | / | + |
| *Megophrys rubrimera* | 26.7–30.5 | / | + | + | + | + | - |
| *Megophrys serchhipii* | 37.1 | / | / | + | / | / | + |
| *Megophrys shapingensis* | 66.0–84.0 | 77.0-104.0 | - | - | + | ++ | +++ |
| *Megophrys shimentaina* | 28.0–30.6 | / | + | + | - | + | + |
| *Megophrys shuichengensis* | 102.0–118.3 | 99.8–115.6 | ++ | - | + | ++ | +++ |
| *Megophrys shunhuangensis* | 30.3–33.6 | 37.6 | + | - | - | - | + |
| *Megophrys stejnegeri* | / | / | ++ | + | / | / | / |
| *Megophrys synoria* | / | / | ++ | / | / | / | / |
| *Megophrys takensis* | 47.3–53.0 | 72.9 | - | + | - | - | + |
| *Megophrys tuberogranulata* | 33.2–39.6 | 50.5 | +or- | - | - | - | + |
| *Megophrys vegrandis* | 27.5–30.6 | / | + | - | + | + | + |
| *Megophrys wawuensis* | 34.4–42.8 | 47.0–49.8 | - | - | + | - | + |
| *Megophrys wugongensis* | 31.0–34.1 | 38.5–42.8 | + | - | - | - | + |
| *Megophrys wuliangshanensis* | 27.3–31.6 | 41.0–41.5 | - | - | +or- | - | - |
| *Megophrys wushanensis* | 30.4–35.5 | 38.4 | - | - | - | -(in female),++(in male) | + |
| *Megophrys xianjuensis* | 31.0–36.3 | 41.6 | + | - | - | - | + |
| *Megophrys xiangnanensis* | 38.6–42.0 | 44.4 | + | - | - | ++ | + |
| *Megophrys yangmingensis* | 33.2–37.1 | 45.2 | + | - | - | + | + |
| *Megophrys zhangi* | 32.5–37.2 | / | - | + | + | + | - |
| *Megophrys zunhebotoensis* | 30.0 | 39.0 | / | + | / | / | / |
